# Granzyme B Is Dispensable in the Development of Diabetes in Non-Obese Diabetic Mice

**DOI:** 10.1371/journal.pone.0040357

**Published:** 2012-07-09

**Authors:** Zia U. Mollah, Kate L. Graham, Balasubramanian Krishnamurthy, Prerak Trivedi, Thomas C. Brodnicki, Joseph A. Trapani, Thomas W. Kay, Helen E. Thomas

**Affiliations:** 1 St. Vincent’s Institute, Fitzroy, Victoria, Australia; 2 Department of Medicine, The University of Melbourne, St. Vincent’s Hospital, Fitzroy, Victoria, Australia; 3 Peter MacCallum Cancer Centre, East Melbourne, Victoria, Australia; Université Paris Descartes, France

## Abstract

Pancreatic beta cell destruction in type 1 diabetes is mediated by cytotoxic CD8^+^ T lymphoctyes (CTL). Granzyme B is an effector molecule used by CTL to kill target cells. We previously showed that granzyme B-deficient allogeneic CTL inefficiently killed pancreatic islets *in vitro*. We generated granzyme B-deficient non-obese diabetic (NOD) mice to test whether granzyme B is an important effector molecule in spontaneous type 1 diabetes. Granzyme B-deficient islet antigen-specific CD8^+^ T cells had impaired homing into islets of young mice. Insulitis was reduced in granzyme B-deficient mice at 70 days of age (insulitis score 0.043±0.019 in granzyme B-deficient versus 0.139±0.034 in wild-type NOD mice p<0.05), but was similar to wild-type at 100 and 150 days of age. We observed a reduced frequency of CD3^+^CD8^+^ T cells in the islets and peripheral lymphoid tissues of granzyme B-deficient mice (p<0.005 and p<0.0001 respectively), but there was no difference in cell proportions in the thymus. Antigen-specific CTL developed normally in granzyme B-deficient mice, and were able to kill NOD islet target cells as efficiently as wild-type CTL *in vitro*. The incidence of spontaneous diabetes in granzyme B-deficient mice was the same as wild-type NOD mice. We observed a delayed onset of diabetes in granzyme B-deficient CD8-dependent NOD8.3 mice (median onset 102.5 days in granzyme B-deficient versus 57.50 days in wild-type NOD8.3 mice), which may be due to the delayed onset of insulitis or inefficient priming at an earlier age in this accelerated model of diabetes. Our data indicate that granzyme B is dispensable for beta cell destruction in type 1 diabetes, but is required for efficient early activation of CTL.

## Introduction

Type 1 diabetes results from destruction of pancreatic beta cells by cytotoxic T cells (CTL). There is evidence from mouse models and human type 1 diabetes that CD8^+^ T cells are the main effectors of beta cell destruction [1]. In non-obese diabetic (NOD) mice, diabetes does not occur in the absence of MHC class I and CD8^+^ T cells [2], [3], [4], [5]. Pathogenic CD8^+^ T cell clones recognizing beta cell autoantigens have been isolated from mice and human subjects with type 1 diabetes [6], [7], [8], [9]. In humans, the majority of cells infiltrating the islets in recently diagnosed patients and in islet grafts undergoing rejection are CD8^+^ T cells [10], [11].

CD8^+^ T cells primarily use perforin and granzymes for destruction of target cells. Perforin is a pore-forming protein that facilitates the entry and function of granzymes in target cells. Granzymes are a family of serine proteases of which there are 11 in mice and 5 in humans [12]. Most granzymes are able to induce cell death *in vitro* in the presence of perforin, however less is known about which granzymes are involved in killing target cells *in vivo*. Granzyme B induces apoptosis in a caspase-dependent manner by cleaving substrates after aspartate residues [13]. It induces activation of the intrinsic apoptosis pathway by cleaving the pro-apoptotic Bcl-2 protein Bid [14], [15], [16], [17]. This leads to mitochondrial membrane permeabilisation by the multi-domain effectors Bax and Bak, release of mitochondrial cytochrome c and activation of the caspase cascade [18], [19]. The pathway downstream of granzyme B has been well characterized in many cell types, including mouse and human pancreatic beta cells [20].

We previously demonstrated that recombinant perforin and granzyme B induce apoptosis of islets *in vitro* [20]. Perforin-deficient NOD mice have a significantly reduced incidence of diabetes [21], [22] and perforin-deficient CTL exhibit significantly reduced cytotoxicity against islet targets *in vitro* compared to wild type CTL [23]. These data indicate the importance of the granule exocytosis pathway in beta cell killing.

While it is clear that perforin is important in development of type 1 diabetes, it is unclear which granzymes participate in perforin-dependent beta cell killing *in vivo*. Granzyme B is a good candidate because it is able to kill beta cells *in vitro* [20], and we previously showed that allogeneic CTL killed beta cells less efficiently in the absence of granzyme B [23], indicating a preferential role for this granzyme in beta cell destruction. To specifically test the role of granzyme B in beta cell destruction in type 1 diabetes, we have backcrossed granzyme B-deficient mice onto the NOD genetic background. We did not observe any effect of granzyme B deficiency on the incidence of diabetes, indicating that there is redundancy in the use of granzymes by CTL to kill target cells. However, we observed a delay in the onset of insulitis in the absence of granzyme B, revealing a role for granzyme B in the initial infiltration of CD8^+^ T cells into islets.

## Results

### Generation of Granzyme B-deficient NOD Mice

Granzyme B-deficient mice on a C57BL/6 background (originally made in 129 embryonic stem cells) [24] were backcrossed onto the NOD genetic background for 10 generations. Backcrossed mice were genotyped for small nucleotide polymorphisms using a 5 K targeted genotyping array. Backcrossed granzyme B-deficient mice were of NOD genotype across the whole genome except for a region on chromosome 14 between, and including, approximately 39.9 (rs13482141) and 61.6 Mb (rs13482212) encompassing the *Gzmb* gene. No known 129 diabetes susceptibility or resistance alleles have been reported for loci within this region [25]. Idd8 (B10 susceptibility allele) and Idd12 (B6 susceptibility allele) both fall outside the genetic interval on chromosome 14 of our backcrossed mice [26].

### Granzyme B-deficient NOD Mice have a Reduced Proportion of Peripheral CD8^+^ T Cells

**Figure 1:**
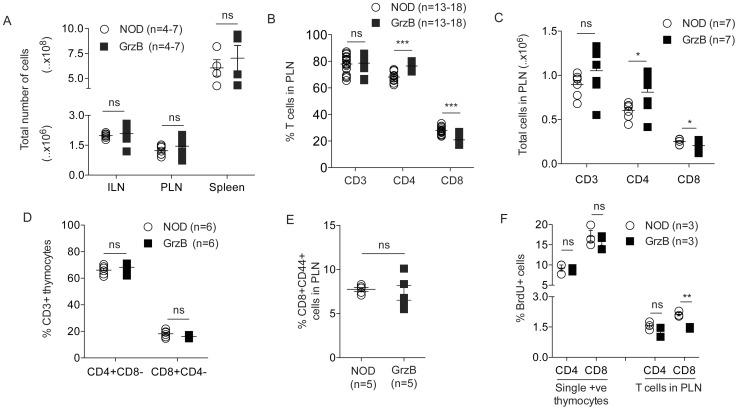
Reduced proportion of CD8^+^ T cells in granzyme B-deficient pancreatic lymph nodes. (A) Total cell counts in ILN, PLN or spleens (n = 4–7, age 70–100 days) from NOD or granzyme B^−/−^ (GrzB) mice. ns = not significant. (B) Pancreatic lymph node cells from 70–100 day old NOD or granzyme B^−/−^ (GrzB) mice were stained with antibodies to CD3 (n = 18), CD4 and CD8 (n = 13). CD3^+^ T cells were gated on CD4 and CD8 to determine the percentage of cells. No difference was observed in the overall percentage of CD3^+^ T cells. The percentage of CD8^+^ T cells was significantly reduced in granzyme B^−/−^ mice compared to NOD (***p = <0.0001), and the percentage of CD4^+^ T cells was increased (***p = <0.0001). Data analyzed by unpaired Student’s t test. (C) Absolute numbers of cells were determined. There was no difference in total CD3^+^ T cells in the PLN of 70–100 day old NOD or granzyme B^−/−^ mice (n = 7 mice). The number of CD4^+^ T cells was increased (n = 7 mice, *p = 0.0442) and the number of CD8^+^ T cells was reduced (n = 7 mice, *p = 0.0421) in the PLN of granzyme B^−/−^ mice. ns = not significant. Data analyzed by unpaired Student’s t test. (D) Percentage of CD4^+^CD8^−^ and CD8^+^CD4^−^ T cells in CD3 gated thymocytes in 70 day old female NOD or granzyme B^−/−^ mice (n = 6 mice performed over 3 independent experiments). ns = not significant. (E) The percentage of CD8^+^ T cells expressing CD44 was determined by flow cytometry at 100 days of age (n = 5). ns = not significant. (F) BrdU incorporation into thymic CD4^+^CD8^−^ and CD8^+^CD4^−^ T cells, and CD4^+^ and CD8^+^ T cells from PLN of 4-week-old NOD and granzyme B^−/−^ mice (n = 3 mice). Percentage of CD8^+^BrdU^+^ cells in PLN was significantly reduced in granzyme B^−/−^ compared to NOD (**p = 0.0012). ns = not significant. Data analyzed by unpaired Student’s t test. Horizontal bars in A-F show mean±SEM.

The total cell counts in the inguinal lymph nodes (ILN), pancreatic lymph nodes (PLN) and spleens were similar in NOD and granzyme B^−/−^ mice at 70 days of age (Fig. 1A). The percentage and number of CD3^+^ T cells in the PLN of NOD and granzyme B^−/−^ mice was similar at 70–100 days of age (Fig. 1B, C), and also in the ILN, spleen and thymus (data not shown). We observed a significantly lower percentage as well as absolute number of CD3^+^CD8^+^ T cells in the peripheral lymphoid organs of granzyme B^−/−^ mice compared to NOD mice (Fig. 1B, C, PLN data shown), and a higher percentage and absolute number of CD3^+^CD4^+^ T cells in granzyme B^−/−^ compared to NOD mice (Fig. 1B, C). The percentage of CD8^+^CD4^−^ and CD4^+^CD8^−^ T cells was similar in the thymus of NOD and granzyme B^−/−^ mice (Fig. 1D). Although there was a decreased number of CD8^+^ T cells, the proportion of these that were activated or effector memory CD8^+^ T cells expressing CD44 was the same in NOD or granzyme B^−/−^ mice at 100 days of age (Fig. 1E). BrdU incorporation into thymic CD4^+^CD8^−^ and CD8^+^CD4^−^ T cells and CD4^+^ T cells in the PLN of NOD and granzyme B^−/−^ mice was similar, but CD8^+^ T cells in the PLN of granzyme B^−/−^ mice showed reduced uptake of BrdU compared to NOD (Fig. 1F).

### Antigen Specific Proliferation of Granzyme B-deficient CD8^+^ T Cells is Reduced

**Figure 2:**
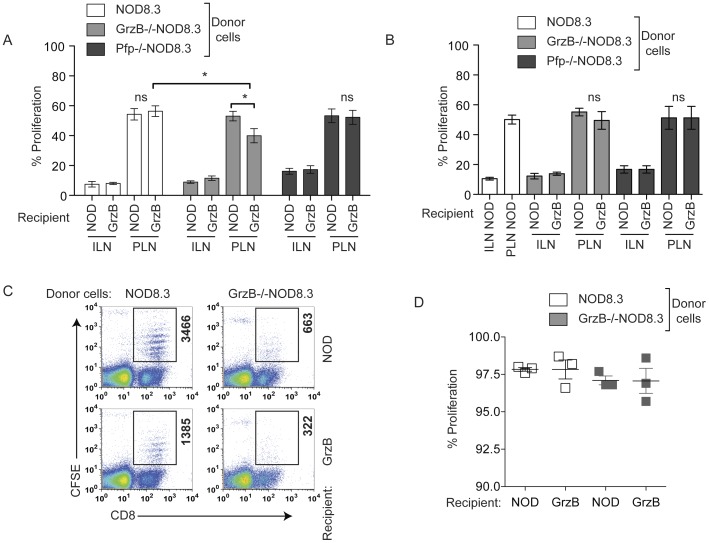
Antigen specific proliferation of granzyme B-deficient CD8^+^ T cells is reduced. (A, B) NOD8.3, GrzB^−/−^NOD8.3 or Pfp^−/−^NOD8.3 splenic CD8^+^ T cells were labeled with CFSE and transferred into 70- or 100-day old female NOD or granzyme B^−/−^ (GrzB) mice. Six days later ILN and PLN were harvested and analyzed for the proliferation of CFSE labeled cells. Data represent mean±SEM of n = 5–10 recipients for NOD8.3 or GrzB^−/−^NOD8.3, n = 4 for Pfp^−/−^NOD8.3, performed as individual mice over at least three independent experiments. Statistical significance, *p = 0.0183 (NOD8.3 vs GrzB^−/−^NOD8.3 in granzyme B^−/−^ PLN) and **p = 0.0315 (GrzB^−/−^NOD8.3 in NOD vs granzyme B^−/−^ PLN). ns = not significant. Data analyzed by unpaired Student’s t test. (C) Dot plots showing proliferation of CFSE labeled NOD8.3 (left panels) or GrzB^−/−^NOD8.3 (right panels) T cells in the islets of 70 day-old female NOD (top panels) or granzyme B^−/−^ (bottom panels) mice. The box in each dot plot shows the number of CD8^+^CFSE^+^ cells that has migrated into the islets. Data are representative of n = 3 experiments. (D) Percentage of CFSE labeled CD8^+^ NOD8.3 or GrzB^−/−^NOD8.3 T cells that have undergone division in the islets of 70 day old NOD or granzyme B^−/−^ recipients (n = 3). No significant difference was observed.

We determined the amount of antigen-specific proliferation of CD8^+^ T cells in the absence of either granzyme B or perforin in 70 or 100 day old mice. To do this we used NOD8.3 mice that have CD8^+^ T cells recognizing the beta cell antigen islet-specific glucose-6-phosphatase catalytic subunit–related protein (IGRP) [8]. We generated NOD8.3 mice deficient in either granzyme B (GrzB^−/−^NOD8.3 mice) or perforin (Pfp^−/−^NOD8.3 mice). Splenic CD8^+^ T cells were labeled with CFSE and transferred intravenously into female NOD or granzyme B^−/−^ mice. Six days later, ILN, PLN or islets were harvested and analyzed for the proliferation of CFSE labeled CD8^+^ T cells. We observed reduced proliferation of GrzB^−/−^NOD8.3 cells in the PLN of 70 day old granzyme B-deficient recipients (Fig. 2A). Proliferation of Pfp^−/−^NOD8.3 CD8^+^ T cells was not different from that of wild-type NOD8.3 cells. No significant differences were observed in antigen specific proliferation at 100 days of age regardless of the presence or absence of granzyme B or perforin (Fig. 2B).

We then examined the ability of IGRP-specific CD8^+^ T cells to migrate to the islets by studying proliferation of CFSE-labeled NOD8.3 or GrzB^−/−^NOD8.3 T cells in the islets. Wild-type NOD8.3 CD8^+^ T cells migrated to and proliferated in 70 day old female NOD or granzyme B^−/−^ islets, however, the migration of GrzB^−/−^NOD8.3 CD8^+^ T cells was inefficient, with less CFSE labeled cells observed in the islets of recipients of these cells (Fig. 2C). Despite the reduced migration of granzyme B-deficient cells, those that were in the islets proliferated to the same extent as wild-type NOD8.3 cells. Figure 2D shows the total percentage proliferation of CFSE labeled cells in the islets. We also observed a similar percentage of cells in each division for NOD8.3 and GrzB−/−NOD8.3 cells (data not shown). We did not observe any difference in activation induced cell death between NOD8.3 and GrzB^−/−^NOD8.3 CD8 T cells *in vitro* (data not shown), indicating that increased cell death is unlikely to account for the reduced presence of granzyme B-deficient cells in the islets.

### Onset of Insulitis is Delayed in Granzyme B-deficient NOD Mice

**Figure 3:**
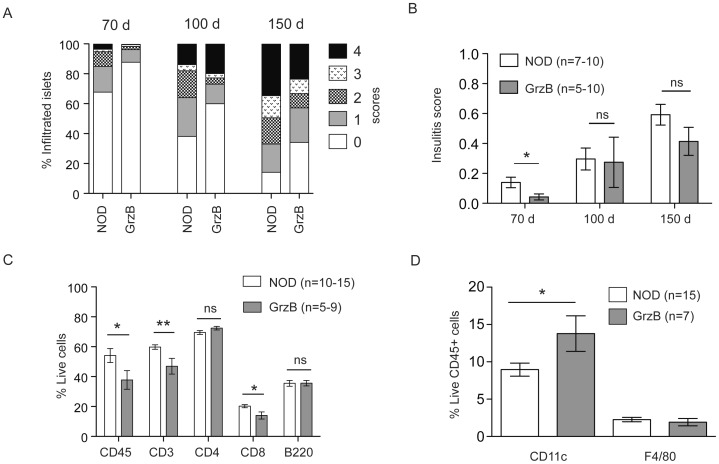
Granzyme B deficient NOD mice have reduced insulitis at 70 days of age, which is not maintained at 100 days. (A) Pancreata from female NOD and granzyme B^−/−^ (GrzB) mice were harvested at 70 days of age (n = 10, total islets scored: NOD = 1004 islets, Granzyme B^−/−^ = 1443 islets), 100 days of age (n = 5–7, total islets scored: NOD = 572 islets, Granzyme B^−/−^ = 548 islets) and 150 days of age (n = 10, total islets scored: NOD = 688 islets, Granzyme B^−/−^ = 1079 islets) and scored for insulitis on frozen sections stained for insulin. The data are the mean of the percentage of islets with each score. (B) For each mouse the insulitis score was calculated as described in material and methods, and data are the mean±SEM insulitis score. Insulitis is significantly reduced in 70 day old granzyme B^−/−^ mice compared to NOD (*p = 0.0274), but not at 100 days or 150 days. Data analyzed by unpaired Student’s t test. ns = not significant. (C) Flow cytometric analysis of islets from female NOD and granzyme B^−/−^ (GrzB) mice at 70 days of age. Live cells were gated using propidium iodide exclusion. CD45 is the proportion of live cells expressing CD45. CD3 is the proportion of live CD45^+^ cells expressing CD3. The proportion of CD4^+^ and CD8^+^ cells was determined as the percentage of live CD45^+^ and CD3^+^ cells. The percentage of CD45^+^ cells (*p = 0.0461), CD3^+^ cells (**p = 0.0087) and CD8^+^ cells (*p = 0.0141) but not CD4^+^ cells was significantly reduced in granzyme B^−/−^ mice. Mean±SEM for n = 10–15 NOD mice and n = 5–9 GrzB mice is shown. 150 islets were used for each sample. Data analyzed by unpaired Student’s t test. (D) The proportion of live CD45^+^ cells in the islets from 70 day old female mice that were CD11c^+^ or F4/80^+^. The percentage of CD11c^+^ cells (*p = 0.0286), but not F4/80^+^ cells (p = 0.5189) were increased in granzyme B^−/−^ mice. Data analyzed by unpaired Student’s t test.

Pancreata from female NOD and granzyme B^−/−^ mice were scored for insulitis at 70 days, 100 days and 150 days of age (Fig. 3A, B). At 70 days of age, insulitis was significantly reduced in granzyme B^−/−^ mice compared to NOD. However, the delay in onset of insulitis in the absence of granzyme B was not maintained, and insulitis in granzyme B^−/−^ NOD mice was the same as wild-type NOD mice at the later time points.

Flow cytometry was performed on islet cells from 70 day old female NOD or granzyme B^−/−^ mice to determine the proportions of infiltrating cells. The percentage of CD45^+^, CD3^+^ and CD8^+^ cells was significantly reduced in islets from granzyme B^−/−^ mice (Fig. 3C). While there was a slight increase in the percentage of CD4^+^ T cells in granzyme B^−/−^ mice, this was not statistically significant (Fig. 3C). The percentage of CD11c^+^ dendritic cells was significantly higher in granzyme B^−/−^ mice compared to NOD whereas no difference was observed in percentage of B220^+^CD45^+^ B cells or F4/80^+^ macrophages (Fig. 3C, D).

### Spontaneous Diabetes in Granzyme B^−/−^ Mice is Similar to NOD

**Figure 4:**
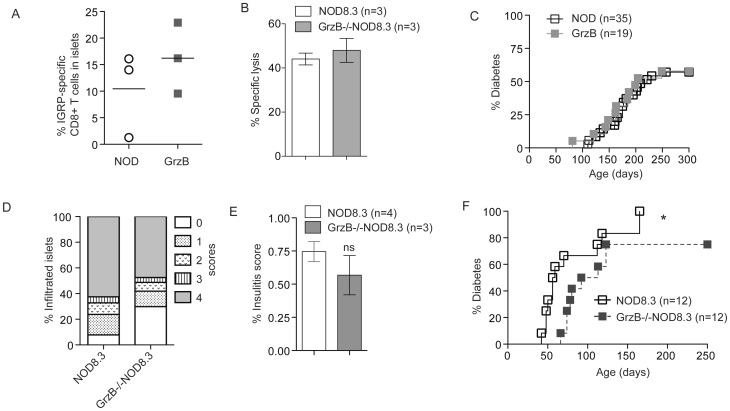
Diabetes is unchanged in granzyme B-deficient NOD mice but is delayed in granzyme B-deficient NOD8.3 mice. (A) The proportion of CD8^+^IGRP-tetramer^+^ T cells in freshly isolated islets from 100 day old female NOD or granzyme B^−/−^ mice was determined by flow cytometry (n = 3). No significant difference was observed. (B) Activated NOD8.3 or GrzB^−/−^NOD8.3 CD8^+^ T cells were cultured for 16 hours with IGRP pulsed and ^51^Cr labeled NOD islets at an effector to target ratio of 20∶1. Data are mean±SEM of three independent experiments. No significant difference was observed. (C) A cohort of female NOD (n = 35) and granzyme B^−/−^ mice (n = 19) mice were observed for 300 days for the incidence of spontaneous diabetes. No difference was observed between the groups. Data analyzed by Log-rank (Mantel-Cox) Test. (D) Pancreata from NOD8.3 or GrzB^−/−^NOD8.3 mice were harvested at 50 days of age for insulitis scoring on insulin antibody-stained frozen sections. Total islets scored: NOD8.3 = 596 islets, GrzB^−/−^ NOD8.3 = 508 islets. (E) For each mouse the insulitis score was calculated using the method as described in material and methods, and data are the mean±SEM insulitis score. Data analyzed by unpaired Student’s t test. ns = not significant. (F) Incidence of diabetes was studied in cohorts of NOD8.3 (n = 12) and GrzB^−/−^NOD8.3 (n = 12) mice. The development of diabetes in GrzB^−/−^NOD8.3 mice was significantly delayed compared to NOD8.3 (*p = 0.0399). Data analyzed by Log-rank (Mantel-Cox) test.

Using tetramer staining, we detected a similar proportion of IGRP-specific CD8^+^ T cells in freshly isolated islets from 100 day old NOD and granzyme B^−/−^ female mice (Fig. 4A). The cytotoxic potential of granzyme B-deficient IGRP-specific CD8^+^ T cells was investigated. Activated CD8^+^ T cells from NOD8.3 or GrzB^−/−^NOD8.3 were able to kill islets from 6 week old NOD mice *in vitro* in a 16 hour ^51^Cr release assay (Fig. 4B). Splenocytes from diabetic NOD (n = 2) or granzyme-B deficient NOD (n = 3) mice were adoptively transferred into RAG1-deficient NOD mice. All recipient mice developed diabetes within 39–53 days of transfer (data not shown). A cohort of female NOD (n = 35) and granzyme B-deficient NOD (n = 19) mice were observed for 300 days for the incidence of spontaneous diabetes. Granzyme B^−/−^ mice developed diabetes at the same rate and incidence as NOD mice (Fig. 4C).

### Deficiency of Granzyme B Delays the Onset of CD8^+^ T Cell-dependent Diabetes

Insulitis scores of female NOD8.3 or GrzB^−/−^NOD8.3 mice at 50 days of age revealed no significant difference (Fig. 4D,E). A cohort of NOD8.3 (n = 12) and GrzB^−/−^NOD8.3 (n = 12) mice was observed for 250 days for development of diabetes. Diabetes was significantly delayed in GrzB^−/−^NOD8.3 mice compared to NOD8.3 mice, and 25% of GrzB^−/−^NOD8.3 mice did not develop diabetes (Fig. 4F). The median age of onset of diabetes in NOD8.3 was 57.5 days compared with 102.5 days in GrzB^−/−^NOD8.3 mice.

## Discussion

Our data demonstrate that granzyme B is dispensable in the development of type 1 diabetes in NOD mice. We previously demonstrated that granzyme B is a marker of cytotoxic potential of CTL, and its expression is increased when CTL enter the islets [27]. In the current study we show that granzyme B may be important for homeostasis of CD8^+^ T cells in the periphery, for efficient proliferation of CD8^+^ T cells in the PLN in the early stages of diabetes pathogenesis, and for homing of primed CTL into the pancreas. However, despite the reduced early expansion, the cytotoxic potential of granzyme B-deficient CTL remains equivalent to that of wild-type CTL.

Only 17% of perforin-deficient NOD mice develop diabetes [22]. In contrast, the incidence of diabetes in the absence of granzyme B was the same as wild-type NOD mice. This difference illustrates the redundancy of perforin-dependent proteases found in the cytotoxic granules of CTL. This redundancy is also apparent in the response of granzyme-deficient mice to viral infection, where mice deficient in individual granzymes have a relatively mild phenotype compared with perforin-deficient animals [28], [29], [30]. It remains unclear which other granzymes are responsible for beta cell killing in the absence of granzyme B, however, granzymes A and M both have reported perforin-dependent cytotoxic activity *in vitro* [12], [31], [32]. We did not observe an increase in granzyme A expression in T cells or NK cells of granzyme B-deficient mice (data not shown), and a compensatory increase in expression of remaining granzymes has not been described in granzyme deficient mice [33]. Perforin-independent mechanisms of beta cell killing, including death receptors (Fas ligand/Fas) and inflammatory cytokines have also been proposed. Our previous data has shown that these mechanisms are of minor importance in NOD mice, and that diabetes still occurs in the absence of these and perforin [34], suggesting that there may be alternative mechanisms of beta cell killing that have not yet been described.

Our data indicate that while the development of single positive CD4^+^ and CD8^+^ T cells in the thymus remained intact in granzyme B-deficient mice, the peripheral pool of CD8^+^ T cells require granzyme B for their expansion, because we observed a reduction in the proportion of CD8^+^ T cells in granzyme B-deficient mice in the spleen, inguinal and pancreatic lymph nodes. Proliferation of granzyme B-deficient antigen-specific CTL was reduced in the pancreatic lymph nodes and migration into the islets was impaired at 70 days of age. However, despite the low frequency of CTL migrating into the islets, those cells reaching the islets proliferated normally. In addition, even though the proportion of CD8^+^ T cells in the periphery was reduced, their ability to develop into activated, islet antigen-specific CTL in the PLN was not affected. Although the mechanism for this remains unclear, it is likely that the reduced proportion of CD8^+^ T cells in the periphery, inefficient priming of CTL in the PLN and reduced homing of CTL into the pancreas accounts for the delay in onset of insulitis in 10 week old mice. It may also explain the delay in diabetes onset in granzyme B-deficient NOD8.3 mice, in which beta cell destruction is dependent on CD8^+^ T cells.

We observed an increased proportion of CD11c^+^ dendritic cells in the islets of granzyme B-deficient NOD mice. It is unlikely that granzyme B plays a role in homeostasis of CD11c^+^ dendritic cells. CD11c^+^ cells in islets present antigen to T cells, but in granzyme B-deficient mice there is a delay in homing of antigen specific CD8^+^ T cells to islets. We speculate that the increased proportion of CD11c^+^ cells is due to the reduced proportion of CD8^+^ T cells at 70 days of age. By 100 days of age, this difference is no longer observed. These changes in proportions of cells in the islets remain inconsequential, however, as by 100 days of age there is no difference in insulitis scores, and the onset and incidence of diabetes in granzyme B-deficient NOD mice is no different from that of wild-type NOD mice.

In conclusion, we have clearly demonstrated a redundancy in granzyme-dependent beta cell killing in type 1 diabetes. This is despite earlier work suggesting that beta cells may be preferentially killed by granzyme B in an allogeneic setting [23]. Our data suggest that while blocking perforin has enormous potential for prevention of CD8^+^ T cell-dependent beta cell killing [22], blocking individual granzymes is of limited value.

## Materials and Methods

### Ethics Statement

All animal studies were conducted at St Vincent’s Institute (Melbourne, Australia) following the guidelines of the institutional animal ethics committee. Animal ethics was approved by the St Vincent’s Hospital Animal Ethics Committee (AEC#016/11).

### Mice

NOD/Lt mice were obtained from the Walter and Eliza Hall Institute animal breeding facility (Kew, Victoria, Australia). Perforin-deficient NOD mice and RAG1-deficient NOD mice were obtained from The Jackson Laboratory (Bar Harbor, USA). Granzyme B-deficient mice were originally made using 129S2/SvPas derived stem cells [24] and then backcrossed onto C57BL/6 for 10 generations. These mice were then backcrossed onto the NOD/Lt genetic background for 10 generations to generate granzyme B-deficient NOD mice. DNA from the 10^th^ generation backcross Granzyme B^−/−^ NOD mice and control NOD/Lt, 129 and C57BL/6 DNA was genotyped by the Australian Genome Research Facility using fluorescently labelled polymorphic markers. Samples were processed for the mouse 5 K targeted genotyping array run on the Affymetrix GeneChip Scanner 3000 7 G MegAllele system. Data was analysed using the GeneChip Targeted Genotyping System software. Regions of difference between strains were identified using data obtained from the Jackson Laboratories Mouse Genome Informatics and NCBI databases.

NOD8.3 mice express the TCRαβ of the H-2K^d^-restricted CD8^+^ T cell clone NY8.3 that recognizes the islet autoantigen islet-specific glucose-6-phosphatase catalytic subunit–related protein (IGRP) [8]. NOD8.3 mice were bred with NOD mice deficient in granzyme B or perforin to generate granzyme B-deficient NOD8.3 mice (GrzB^−/−^NOD8.3) or perforin-deficient NOD8.3 mice (Pfp^−/−^NOD8.3).

### Histology and Insulitis Scoring

Pancreata were dissected from 70, 100 and 150-day-old female wild-type or granzyme B^−/−^NOD mice and 50-day-old female NOD8.3 and GrzB^−/−^NOD8.3 mice, and frozen in OCT (Optimal Cutting Temperature) compound (Tissue-Tek®, Sakura). 5 μm cryostat sections were cut at three levels, 200 μm apart. Sections from the three levels were stained with guinea pig anti-insulin Ab (Dako Cytomation, CA) followed by horse radish peroxidase-anti-guinea pig Ig (Dako Cytomation). Insulitis scoring was performed in a blinded manner based on the following criteria: score 0, no infiltrate; score 1, peri-islet infiltrate; score 2, extensive peri-islet infiltrate; score 3, intra-islet infiltrate and score 4, extensive intra-islet infiltrate or total beta-cell loss.

We further analyzed the percentage of insulitis using the method previously described [35]. For each mouse the insulitis score was calculated as (number of islets at score 1×0.25+ number of islets at score 2×0.50+ number of islets at score 3×0.75+ number of islets at score 4×1.00)/total number of islets.

### Diabetes Monitoring

NOD, granzyme B^−/−^, NOD8.3 and GrzB^−/−^NOD8.3 mice were monitored for spontaneous diabetes by urinary glucose measurement using Diastix (Bayer diagnostics, Bridgend, UK). Diabetes was confirmed by blood glucose measurement using Advantage II Glucose Strips (Roche, Basel, Switzerland). Mice with blood glucose >18 mM on consecutive days were considered diabetic. For adoptive transfer of diabetes, 2×10^7^ splenocytes from newly diabetic NOD or granzyme B^−/−^ female mice were transferred i.v. into 8 week old RAG1-deficient NOD female mice. Mice were monitored for diabetes as described above.

### Islet Isolation and Flow Cytometry

Islets were isolated as previously described [36]. For flow cytometry, freshly isolated islets were dispersed into single cell suspensions as previously described [36]. The cells were allowed to recover for 1 hour at 37°C and then stained using standard procedures. Fluorescently conjugated specific antibodies were used to analyze T cells (anti-CD3 FITC, BD Pharmingen), CD4^+^ T cells (anti-CD4 PE, BioLegend), CD8^+^ T cells (anti-CD8 APC, BioLegend), dendritic cells (anti-CD11c FITC, BD Pharmingen), macrophages (anti-F4/80 APC, Caltag) and B cells (anti-B220 PE, BD Pharmingen) on leukocytes (anti-CD45 PerCp, BD Pharmingen). When anti-CD45 PerCp was used, propidium iodide was used at 3 µg/ml. Beta cells were identified as CD45-negative autofluorescent cells as previously described [37]. For the detection of islet infiltrating IGRP-specific T cells, freshly isolated islets from 100 days old mice were stained with PE-conjugated H-2K^d^ IGRP_206–214_ tetramers (ImmunoID, Melbourne, Australia) and APC-conjugated anti-CD8 Ab. All analysis was performed on a FACSCalibur (Becton Dickinson, Franklin Lakes, NJ), and using FlowJo software (Treestar, Ashland, OR).

Spleens, thymus, inguinal and pancreatic lymph nodes were dissected from 70–100 day old female mice, treated with 0.02% Collagenase P (Roche Diagnostics, GmBH) to make single cell suspensions and counted. For spleens, red blood cells were lysed with 0.747% ammonium chloride before counting. Cells were stained with fluorescently conjugated anti-CD3, anti-CD4 and anti-CD8 antibodies and analyzed by flow cytometry. The absolute cell counts were determined using the percentage of CD3^+^, CD4^+^ or CD8^+^ cells and the total cell numbers.

### *In vivo* Proliferation Assay

4-week-old female NOD and granzymeB^−/−^ mice were injected with 3 mg of BrdU (Sigma-Aldrich, St Louis, MO) in 200 µl PBS intraperitonially. Thymus and pancreatic lymph nodes were harvested 24 hours after the injection. Cells were stained with antibodies to CD4, CD8 and BrdU (APC BrdU Flow Kit, BD Pharmingen) and analyzed by flow cytometry.

### Transfer of IGRP-specific T Cells

Splenic CD8^+^ T cells from 8–10 week old female NOD8.3, GrzB^−/−^NOD8.3 or Pfp^−/−^NOD8.3 mice were labeled with 5 μM carboxyfluorescein succinimidyl ester (CFSE), and transferred i.v. into 70 day and 100 day old female mice. After 6 days, pancreatic lymph nodes (PLN), inguinal lymph nodes (ILN) and islets were analyzed by flow cytometry for proliferation of donor CD8^+^ T cells.

### ^51^Cr Release Assay

^51^Cr release assays were performed as previously described [38]. Islets from 6 week old male NOD mice were loaded with 200 mCi [^51^Cr] sodium chromate (Amersham Pharmacia Biotech, Piscataway, NJ) for 90 min, then plated in a U-bottomed 96 well plate at 10 islets/well. P815 mastocytoma cells loaded with specific IGRP_206–214_ peptide and ^51^Cr were used as a positive control. Target cells were incubated with *in vitro*-activated NOD8.3 or GrzB^−/−^NOD8.3 CD8+ T cells at a 20∶1 effector:target ratio in triplicate for 16 h at 37°C. Medium alone or 2% TRITON X-100 was added to a set of target cells to determine spontaneous and total cell lysis, respectively. The radioactivity of harvested supernatant was measured on a gamma counter (Perkin-Elmer). Specific ^51^Cr release was calculated as percent lysis = (test cpm – spontaneous cpm)/(total cpm – spontaneous cpm) ×100.
